# Serum Glycoproteomic Alterations in Patients with Diabetic Retinopathy

**DOI:** 10.3390/proteomes8030025

**Published:** 2020-09-13

**Authors:** Ashok Sharma, James Cox, Joshua Glass, Tae Jin Lee, Sai Karthik Kodeboyina, Wenbo Zhi, Lane Ulrich, Zachary Lukowski, Shruti Sharma

**Affiliations:** 1 Center for Biotechnology & Genomic Medicine, Augusta University, Augusta, GA 30912, USA; assharma@augusta.edu (A.S.); JAMCOX@augusta.edu (J.C.); JOSGLASS@augusta.edu (J.G.); TALEE@augusta.edu (T.J.L.); SKODEBOYINA@augusta.edu (S.K.K.); WZHI@augusta.edu (W.Z.); 2 Department of Ophthalmology, Augusta University, Augusta, GA 30912, USA; LULRICH@augusta.edu (L.U.); ZLUKOWSKI@augusta.edu (Z.L.); 3 Department of Population Health Sciences, Augusta University, Augusta, GA 30912, USA; 4 Culver Vision Discovery Institute, Augusta University, Augusta, GA 30912, USA

**Keywords:** diabetic retinopathy, glycoproteomics, LC–MS/MS

## Abstract

The precise molecular mechanisms of diabetic retinopathy (DR) pathogenesis are unclear, and treatment options are limited. There is an urgent need to discover and develop novel therapeutic targets for the treatment of this disease. Glycosylation is a post-translational modification that plays a critical role in determining protein structure, function, and stability. Recent studies have found that serum glycoproteomic changes are associated with the presence or progression of several inflammatory diseases. However, very little is known about the glycoproteomic changes associated with DR. In this study, glycoproteomic profiling of the serum of diabetic patients with and without DR was performed. A total of 15 glycopeptides from 11 glycoproteins were found to be significantly altered (5 upregulated and 10 downregulated) within the serum glycoproteome of DR patients. These glycoproteins are known to be involved in the maintenance of the extracellular matrix and complement system through peptidolytic activity or regulation.

## 1. Introduction

Diabetic retinopathy (DR) is a microvascular complication of diabetes and remains the primary cause of permanent blindness in the working age population [1]. The prevalence of DR is expected to increase due to the global uptrend of both diabetes mellitus and life expectancy [2]. The precise molecular mechanisms of DR pathogenesis are not clear, and treatment options are limited [3]. Additionally, detection of this disease at an early stage is very difficult mainly due to a late onset of irreversible symptoms [4]. There is an urgent need to discover novel therapeutic targets and develop early detection methods for the treatment of DR.

Glycosylation is a common post-translational modification (PTM), in which an oligosaccharide (glycan) is enzymatically attached to a protein at either an asparagine (N) residue (N-linked) or a serine (S)/threonine (T) residue (O-linked). Glycosylation plays a critical role in determining protein structure, function, and stability. Glycoproteins participate in the regulation of a variety of cellular and biological functions, including cell growth, adhesion, signaling, and inflammation [5]. Changes in glycosylation patterns of systemic proteins have been observed in inflammation, immune deficiencies, cancers, and autoimmune diseases [6,7,8,9,10,11]. Additionally, in the case of chronic inflammatory conditions, acute phase proteins, transport proteins, and proteins related to the complement system were found to contain altered glycosylation patterns [12]. However, studies exploring the serum glycoproteomic profiles in the context of DR are limited.

In this study, we performed comprehensive glycoproteomic profiling of serum samples from diabetic patients with and without DR using LC–MS/MS technology. Significant differences were found in the glycosylation patterns in the serum of DR patients. Discovery of altered glycosylation profiles associated with DR opens a new area of research for DR diagnosis and surveillance.

## 2. Materials and Methods

### 2.1. Human Subjects and Sample Collection

This study was approved by the Institutional Review Board (IRB) of the Augusta University, Augusta, Georgia (IRB#1458143). Intravenous peripheral blood samples from 23 type-2 diabetic patients, 10 with DR (cases) and 13 without DR (controls), were collected after obtaining informed consent. The patients with other diabetic complications including peripheral neuropathy, diabetic foot, and chronic kidney disease were excluded from this study. The demographics of the patient population used in the study are presented in Table 1. Approximately 10 mL of venous blood was collected from each consenting patient in a serum separator tube (Vacutainer SST; Catalog#BD367985; BD Biosciences, San Jose, CA, USA). Blood samples were allowed to clot for 30 min and subsequently centrifuged at 1300× *g* in a fixed angle centrifuge for 15 min. The serum (approximately 5 mL) was then isolated and immediately stored at −80 °C until further studies.

### 2.2. Sample Preparation and Digestion

Analysis of serum proteins is often masked by the presence of high concentrations of albumin and IgG that can make up a large proportion (>70%) of total serum protein. These proteins were depleted, to improve the dynamic range and detection of low-abundant proteins, using a spin depletion cartridge (Multiple Affinity Removal Spin Cartridge Human 14, MARS-14, Agilent, Santa Clara, CA, USA). The depleted serum samples were lyophilized and reconstituted into 40 µL of 8 M urea in 50 mM Tris-HCl (pH 8). Reduction and alkylation of the cysteine residues were then performed with 20 mM DTT and 55 mM iodoacetamide, respectively, followed by adding 360 µL of 50 mM ammonium bicarbonate buffer to reduce the urea concentration to below 1 M. Protein concentration of the samples was measured using a Bradford Assay Kit according to the manufacturer’s instructions (Pierce, Rockford, IL, USA). Trypsin (Pierce, Rockford, IL, USA) was then added to 50 µg protein from each sample at a 1:20 ratio (*w*/*w*) to perform protein digestion at 37 °C overnight.

### 2.3. LC–MS/MS Analysis

Digested serum samples were first cleaned using a C18 spin plate (Nest Group, Southborough, MA, USA) and then analyzed using an Orbitrap Fusion tribrid mass spectrometer (Thermo Scientific, Waltham, MA, USA) coupled with an Ultimate 3000 nano-UPLC system (Thermo Scientific). Two micrograms of reconstituted peptide (1 µg/mL) was first trapped and washed on a Pepmap100 C18 trap (5 μm, 0.3 × 5 mm) at 20 μL/min using 2% acetonitrile in water (with 0.1% formic acid) for 10 min and then separated on a Pepman100 RSLC C18 column (2.0 μm, 75 μm × 150 mm) using a gradient of 2 to 40% acetonitrile with 0.1% formic acid over 120 min at a flow rate of 300 nL/min and a column temperature of 40 °C. Eluted peptides were introduced into an Orbitrap Fusion MS via nano-electrospray ionization source with a temperature of 300 °C and spray voltage of 2000 V. The peptides were analyzed by data-dependent acquisition in positive mode using an Orbitrap MS analyzer for a precursor scan at 120,000 FWHM from 400 to 2000 *m*/*z* and an ion-trap MS analyzer for MS/MS scans in top speed mode (3 s cycle time) with dynamic exclusion settings (repeat count 1 and exclusion duration 15 s). Higher-energy collisional dissociation (HCD) was used as a fragmentation method with a normalized collision energy of 32%.

### 2.4. Protein Identification and Quantification

Identification and quantification of glycopeptides were performed using Byonic software (v3.7.13, Protein Metrics, Cupertino, CA, USA) against the UniProt human protein database (reviewed and annotated UniProt Knowledge Human Database; Release August 2017) with the following settings: fully specific trypsin digestion, one missed cleavage site, precursor mass tolerance of 10 ppm, fragmentation type of QTOF/HCD, fragment mass tolerance of 0.5 Da, fixed carbidomethylation (+57.021 Da) for cysteine, dynamic oxidation (+15.995 Da) for methionine, and 2% FDR. N- and O-glycans were searched against their respective glycan databases. Total modifications per peptide were set to 3 with a maximum of 2 common modifications and 1 rare modification.

### 2.5. Statistical and Bioinformatics Analysis

All statistical analyses were conducted using the R Project for Statistical Computing (v3.2.5, R Core Team, Vienna, Austria). Peptide-spectrum match (PSM) count data for the identified glycopeptides were log2 transformed, and differential expression analyses were conducted using the *“edgeR”* (v3.26.8) package [13]. FDR adjusted *p*-value < 0.05 was considered significant. Gene ontology enrichment analysis was conducted to identify biological processes, cellular components, and molecular functions associated with the identified serum glycoproteins. The “goana” function from “limma” (v3.40.6) package was used to perform over-representation analyses for gene ontology terms [14]. The *p*-values were adjusted for multiple testing using the FDR method.

## 3. Results

### 3.1. Glycosylation Profiling of Human Serum

**Figure 1:**
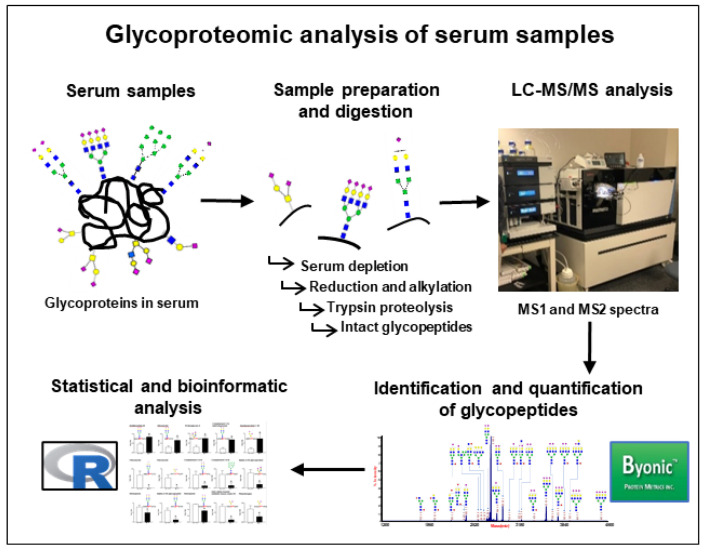
Workflow of glycoproteomic analysis to identify and quantify glycopeptides in serum of patients with and without diabetic retinopathy (DR). After depletion of the most abundant serum proteins, reduction and alkylation of the cysteine residues were performed with DTT and iodoacetamide, respectively. Protein digestion was performed using trypsin, and digested samples were analyzed using an Orbitrap Fusion tribrid mass spectrometer coupled with an Ultimate 3000 nano-UPLC system. Identification and quantification of glycopeptides was performed using Byonic software, and statistical analyses were performed using R-software.

**Figure 2:**
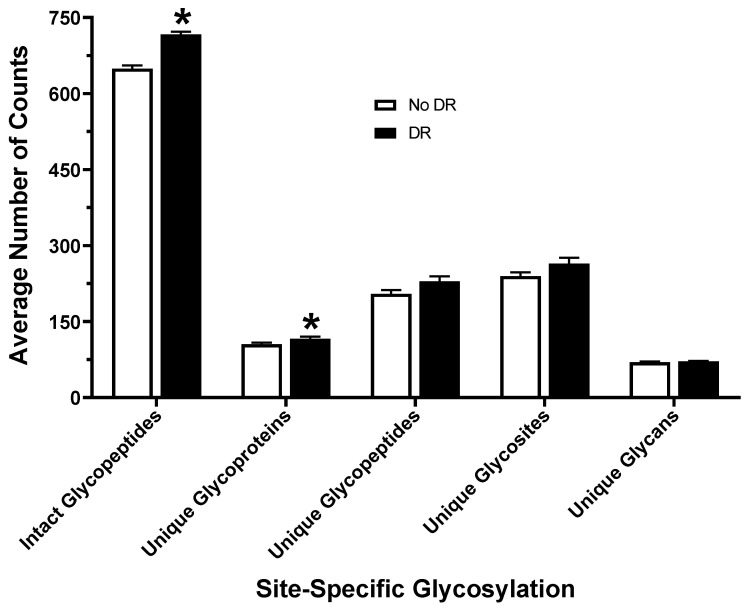
Number of total and unique glycopeptides, unique glycoproteins, glycosites, and glycans identified in the serum from diabetic patients with and without DR. * *p*-value < 0.05.

After protein digestion, samples were analyzed via LC–MS/MS, and Byonic software was used to identify intact glycopeptides and sites of glycosylation on glycoproteins in all 23 serum samples, as shown in Figure 1. Overall, the average number of total glycopeptides detected in serum samples from patients with DR (717.0 ± 5.0) was significantly higher than in patients without DR (648.9 ± 6.8) (Figure 2). The number of unique glycoproteins detected per sample was also significantly higher in patients with DR (116.1 ± 4.1) as compared to patients without DR (105.2 ± 3.2). There was no difference in the number of unique glycopeptides (229.3 ± 9.8 vs. 204.8 ± 7.4), unique sites of glycosylation (264.6 ± 11.5 vs. 239.6 ± 7.8), or unique glycans (71.5 ± 0.9 vs. 69.5 ± 1.4) found in serum of DR samples as compared to those without DR (Figure 2). The most abundant glycopeptides detected in human serum are listed in Table 2. Hemopexin (HPX), alpha-2-HS-glycoprotein (AHSG), inter-alpha-trypsin inhibitor heavy chain H1 (ITIH1), complement factor H (CFH), ceruloplasmin (CP), haptoglobin (HP), inter-alpha-trypsin inhibitor heavy chain H4 (ITIH4), complement C4-B (C4B), prothrombin (F2), plasma protease C1 inhibitor (SERPING1), beta-2-glycoprotein 1 (APOH), heparin cofactor 2 (SERPIND1), and antithrombin-III (SERPINC1) were found to be the most prevalent glycoproteins in the human serum. Among the glycans, HexNAc(1)Hex(1)NeuAc(1) (1.1.0.1) and HexNAc(4)Hex(5)NeuAc(2) (4.5.0.2) were the two most prevalent detected on the human serum glycoproteins (Table 2).

### 3.2. N-Glycans versus O-Glycans

**Figure 3:**
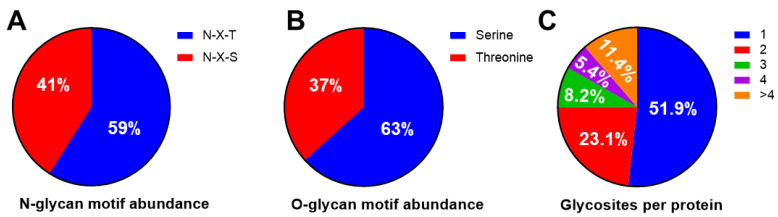
Relative abundance of typical N- and O- glycan sequence motifs and glycosites per protein detected in serum samples. (**A**) N-linked glycans have a consensus sequence N-X-S or N-X-T (where X denotes any amino acid except proline). In analysis of serum samples, the prevalence of N-glycan sequence motifs, N-X-T, was 59%, whereas the prevalence of N-X-S was 41%. (**B**) Among O-glycans, 63% were linked to a serine (S) residue, and 37% were linked to a threonine (T) residue. (**C**) Glycoproteins can have more than one site of glycosylation. The percentages of glycoproteins with 1, 2, 3, 4, >4 glycosite(s) are 51.9%, 23.1%, 8.2%, 5.4%, and 11.4%, respectively.

N-linked glycans have a core containing two GlcNAc residues and three mannose residues attached to the asparagine (N) at the consensus sequence N-X-S/T (where X denotes any amino acid except proline). O-glycans have the linkage of an N-acetylgalactosamine (GalNAc) moiety to the hydroxyl of serine (S) or threonine (T). Among total N-glycans detected in the serum, the N-X-T binding motif was more common than the N-X-S motif (59% vs. 41%) (Figure 3A), whereas among the O-glycans, 63% were linked to a serine residue, and 37% were linked to a threonine residue (Figure 3B). Glycoproteins have one or more sites of glycosylation. In our study, a total of 51.9% of glycoproteins were found to be glycosylated at one site, while 23.1%, 8.2%, 5.4%, and 11.4% of glycoproteins were glycosylated at two, three, four, and more than four glycosites, respectively (Figure 3C).

**Figure 4:**
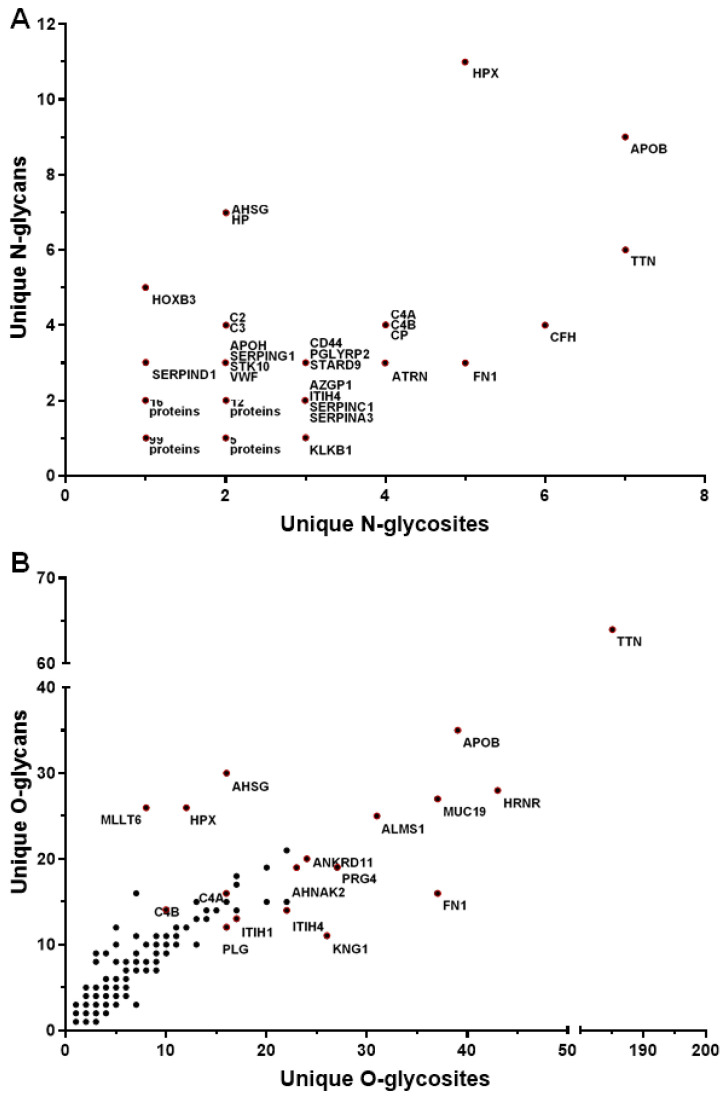
The distribution of unique glycans and glycosites on the serum glycoproteins. For each glycoprotein, the x-axis represents the number of glycosites, and the y-axis represents the number of unique glycans. The scatterplots depicting the distribution of unique N-linked glycans and glycosites and unique O-linked glycans and glycosites detected in serum glycoproteins are shown in panels (**A**) and (**B**), respectively.

A summary of the number of glycosites and the number of unique glycans for each N-glycoprotein found in all serum samples is shown in Figure 4A. The glycoproteins with the most unique N-glycosites were apolipoprotein B-100 (APOB, 7 glycosites, 9 glycans), titin (TTN, 7 glycosites, 6 glycans), complement factor H (CFH, 6 glycosites, 4 glycans), fibronectin (FN1, 5 glycosites, 3 glycans), hemopexin (HPX, 5 glycosites, 11 glycans), attractin (ATRN, 4 glycosites, 3 glycans), ceruloplasmin (CP, 4 glycosites, 4 glycans), complement factor 4A (C4A, 4 glycosites, 4 glycans,) complement C4-B (C4B, 4 glycosites, 4 glycans), antithrombin-III (SERPINC1, 3 glycosites, 2 glycans), alpha-1-antichymotrypsin (SERPINA3, 3 glycosites, 2 glycans), and haptoglobin (HP, 2 glycosites, 7 glycans). Glycoproteins found to have the most O-glycosites were titin (TTN, 185 glycosites, 64 glycans), hornerin (HRNR, 43 glycosites, 28 glycans), apolipoprotein (APOB, (39 glycosites, 35 glycans), fibronectin (FN1, 37 glycosites, 16 glycans), mucin-19 (MUC19, 37 glycosites, 27 glycans), alstrom syndrome protein (ALMS1, 31 glycosites, 25 glycans), proteoglycan 4 (PRG4, 27 glycosites, 19 glycans), kinonogen-1 (KNG1, 26 glycosites, 9 glycans), inter-alpha-trypsin inhibitor heavy chain H4 (ITIH4, 22 glycosites, 14 glycans), inter-alpha-trypsin inhibitor heavy chain H1 (ITIH1, 17 glycosites, 13 glycans), plasminogen (PLG, 16 glycosites, 12 glycans), and alpha-2-HS glycoprotein (AHSG, 16 glycosites, 30 glycans) (Figure 4B). These findings are in agreement with current literature, as the O-glycoproteome features more heterogeneity than the N-glycoproteome. This may be due to O-glycans having a simpler structure compared to N-glycans. Additionally, there are two amino acid residues (S and T) where O-glycans are linked versus the one amino acid (N) in the case of N-glycans.

### 3.3. Gene Ontology Enrichment Analyses of Glycoproteins Detected in Serum

**Figure 5:**
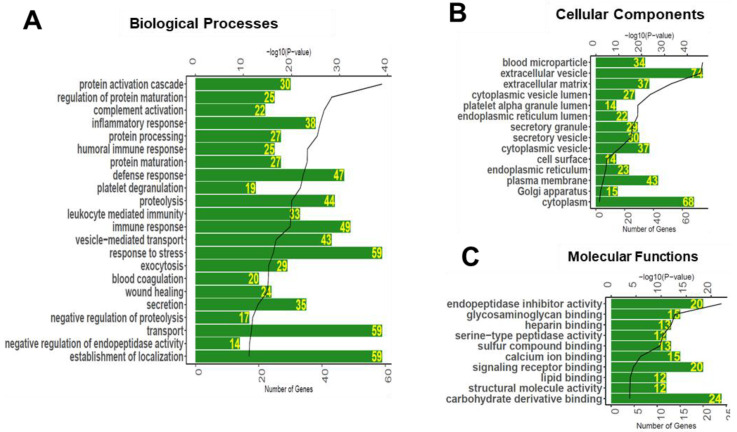
Biological processes (**A**), cellular compartments (**B**), and molecular functions (**C**) associated with the glycoproteins detected in the human serum. Bioinformatics analysis was performed to associate significantly enriched Gene Ontology (GO) terms to the serum glycoproteins. The green bar represents the number of proteins annotated to each GO term, and black line represents the *p*-value of enrichment.

Gene ontology (GO) enrichment analyses were performed to annotate the detected serum glycoproteins with biological processes, molecular functions, and cellular compartments (Figure 5). The biological functions enriched in the serum glycoproteins were found to include protein activation cascade, regulation of protein maturation, complement activation, inflammatory response, leukocyte mediated immunity, vesicle-mediated transport, response to stress, exocytosis, blood coagulation, wound healing, secretion, endopeptidase activity, and establishment of localization (Figure 5A). As expected, the cellular components enriched in the serum glycoproteins were found to include blood microparticle, extracellular vesicle, extracellular matrix, secretory vesicle, cytoplasmic vesicle, cell surface, endoplasmic reticulum, Golgi apparatus, and cytoplasm (Figure 5B). The molecular functions enriched in the serum glycoproteome included endopeptidase inhibitor activity, glycosaminoglycan binding, heparin binding, serine-type peptidase activity, sulfur compound binding, signaling receptor binding, lipid binding, structural molecule activity, and carbohydrate derivative binding (Figure 5C).

### 3.4. Serum Glycoproteomic Alterations Associated with Diabetic Retinopathy

**Figure 6:**
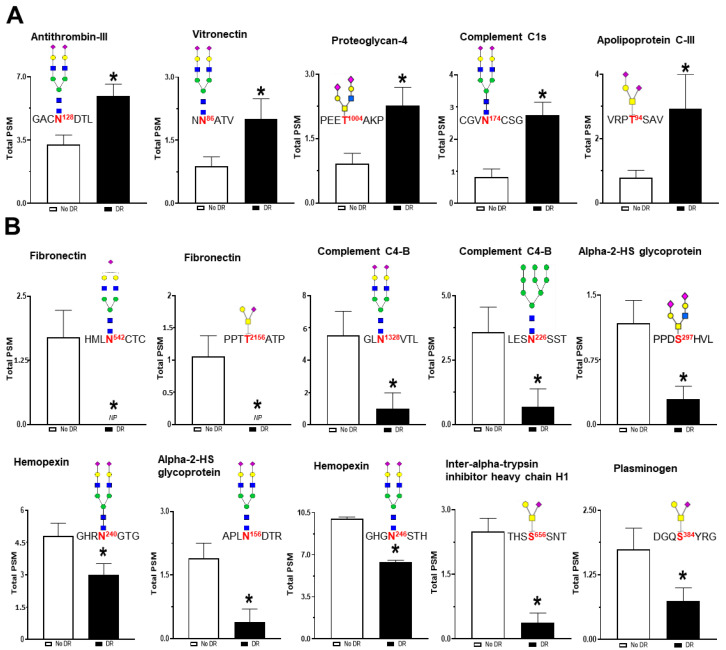
The difference in the glycopeptide levels in the serum of patients with and without DR. Each bar represents the PSM (peptide spectrum match) values of the glycopeptides detected in the serum samples. The site of glycosylation and the structure of the glycan attached are also shown for each peptide. Statistical analysis revealed that levels of five glycopeptides were significantly upregulated (**A**), whereas the levels of 10 glycopeptides were significantly downregulated (**B**). * *p*-value < 0.05.

The quantification of glycopeptides was performed by counting peptide-spectrum match (PSM) values. PSM values for each peptide indicate the total number of mass spectra associated with each peptide. Statistical analysis revealed 15 glycopeptides significantly altered in the serum of patients with DR as compared to those without DR (Table 3). These 15 glycopeptides belong to 11 glycoproteins including apolipoprotein C-III (APOC3), complement C1s subcomponent (C1S), proteoglycan 4 (PRG4), vitronectin (VTN), antithrombin-III (SERPINC1), fibronectin (FN1), complement C4-B (C4B), alpha-2-HS-glycoprotein (AHSG), hemopexin (HPX), inter-alpha-trypsin inhibitor heavy chain H1 (ITIH1), and plasminogen (PLG). The significantly upregulated glycopeptides within DR serum belong to the glycoproteins APOC3 (T-94; 3.21-fold), C1S (N-174; 2.86-fold), PRG4 (T-1004; 2.21-fold), VTN (N-86; 2.10-fold), and SERPINC1 (N-128; 1.79-fold). Glycopeptides that are downregulated in DR belong to the following glycoproteins: FN1 (N-542; 0.069-fold and T-2156; 0.107-fold), C4B (N-226; 0.207-fold and N-1328; 0.185-fold), AHSG (N-156; 0.641-fold and S-297; 0.328-fold), HPX (N-240; 0.256-fold and N-246; 0.639-fold), ITIH1 (S-656; 0.193-fold), and PLG (S-384; 0.468-fold). The bar plots depicting the glycopeptide levels detected in serum of patients with and without DR are shown in Figure 6.

### 3.5. Trends in Sialylation and Fucosylation of Glycans

**Figure 7:**
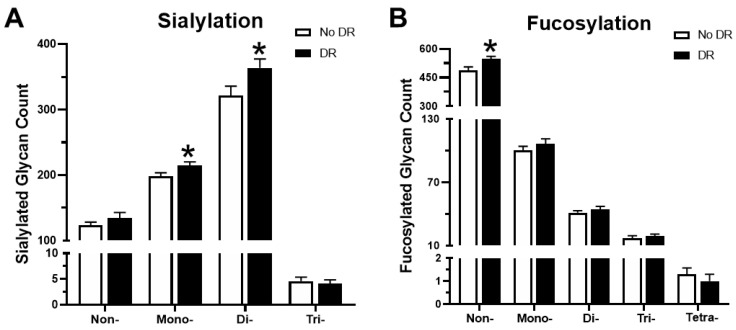
Variations in sialylated and fucosylated glycopeptides in the serum. (**A**) The number of non- sialylated, and mono-, di-, and tri-sialylated glycan structures in patients with and without DR. (**B**) Fucosylation trends between the two groups. * *p*-value < 0.05.

The terminal sugar residues of glycans such as sialic acid and fucose participate in cell–cell interactions and play an important role in the function of glycoproteins. Changes in the sialylation and fucosylation of glycopeptides in the serum were also investigated. There was an 8.3% increase in mono-sialylated glycopeptides and a 13.2% increase in di-sialylated glycopeptides in patients with DR as compared to patients without DR, whereas no significant differences were observed in non-sialylated or tri-sialylated glycopeptides (Figure 7A). Analysis of fucosylation revealed that non-fucosylated glycopeptides were significantly increased (12% increase) in patients with DR, and there was no significant difference in mono-, di-, tri-, and tetra-fucosylated glycopeptides (Figure 7B).

## 4. Discussion

Recent studies have shown the significance of serum glycoproteomic changes in several diseases [15,16,17]. However, little information is available regarding the relevance of the serum glycoproteome in the pathogenesis of DR. In this study, using LC–MS/MS, both N- and O-linked intact glycopeptides were identified and quantified in the serum from patients with and without DR. Therefore, this study provides a comprehensive site-specific glycosylation map of serum glycoproteins and glycosylation changes associated with DR. On average, 229 unique glycopeptides from 116 glycoproteins were detected in each serum sample. A total of 15 distinct glycopeptides from 11 glycoproteins displayed altered glycosylation patterns in the DR patients. These glycopeptides belong to proteins involved in a host of functions, including protease regulation, inflammatory effects, cell adhesion, maintenance of the extracellular matrix (ECM), and the complement activation pathway. These pathways are known to play important roles in the pathogenesis of DR.

Two glycopeptides of fibronectin (sites N-542 and T-2156) showed changes in glycosylation in patients with DR. The expression of fibronectin in the retina, plasma, and kidneys has been shown to be increased in diabetes and has been associated with inflammation and retinal dysfunction [18,19,20]. Fibronectin is involved in ECM remodeling via cell-adhesion, migration, and thrombosis regulation [18]. In our study, we found decreased glycosylation of this protein at both sites. Previous studies have shown that the N-linked oligosaccharides of fibronectin act as modulators of biological functions of the glycoprotein, and the lack of carbohydrates significantly increases its ability to promote adhesion and spreading of fibroblasts [21]. N-glycans on fibronectin regulate cell adhesive capability by modulating cell-fibronectin interactions [22].

Proteoglycan 4 (PRG4), also known as lubricin, is a lubricating mucin-like glycoprotein that helps in reducing shear at the cartilage surface. Although the main function of this glycoprotein is lubrication for joints, it has been found to play a protective role at the ocular surface with functions as a boundary lubricant [23]. We found increased glycosylation of PRG4 at site T-1004 in patients with DR. Recent studies have shown inhibitory effects of PRG4 on the migration and proliferation of human venous cells as well as its protective role in preventing damage to the corneal epithelium [24,25]. Additionally, it has been shown that glycosylation of this protein is responsible for recruitment of leucocytes to inflammatory sites [26].

Two proteins within the complement system (C4B, C1S) displayed altered glycosylation patterns in DR. The levels of glycosylation of C4B were decreased at sites N-226 and N-1328, while C1S showed increased glycosylation at site N-174. C4B is a non-enzymatic molecule of complement C4 that is produced when C4 is proteolytically cleaved. C4B acts similar to an antibody, which binds to the surface of pathogens to mark them for phagocytosis [27]. Additionally, C4B has been linked to DR pathology in previous studies [28]. C1S is a serine protease that cleaves and activates the C1 complex, triggering the complement cascade involved in immune responses [29]. Emerging evidence has shown activation of the complement system to be involved in vascular abnormalities associated with DR [30].

Our analysis revealed that several altered glycoproteins, including fibronectin, antithrombin-III, alpha-2-HS-glycoprotein, inter-alpha-trypsin inhibitor heavy chain H1, hemopexin, and vitronectin are involved in peptidolytic processes through regulatory effects [18,31,32,33,34,35]. The altered glycosylation of serum peptidase regulators may offer novel insights into the pathogenesis of DR. ITIH1, a peptidase inhibitor, has previously been identified to be a potential biomarker of DR [36]. In our study, we found that glycosylation of ITIH1 was attenuated in the serum of patients with DR at site S-656. Glycosyl modification of ITIH1 has been shown to produce a physical barrier between insulin receptors and circulating insulin, exacerbating insulin resistance and subsequently increasing inflammation [37]. AHSG is another glycoprotein that exhibits peripheral insulin resistance via protease regulation [32]. Elevated serum AHSG levels have been found in patients with DR and associated with angiogenesis and inflammation [38]. SERPINC1 is a serine protease inhibitor involved in dampening the coagulation cascade that has been observed to provide anti-inflammatory effects to the vascular endothelium by interacting with heparin-like substances [39]. It has been documented that SERPINC1 concentration is normal in the serum of diabetics but shows an inverse correlation between non-enzymatic glycosylation (i.e., glycation) levels and function [40]. Our study found that SERPINC1 was more glycosylated in serum of patients with DR, likely leading to unchecked inflammatory effects on vascular walls.

The interactions of vitronectin and fibronectin with heparin-like substances play a large part in the coagulation cascade and contain novel regulatory functions. Fibronectin has been shown to be involved in the regulation of vascular endothelial-growth factor (VEGF) through a heparin-induced model [41]. Vitronectin also interacts with heparin in circulation and, after interaction, has been shown to oligomerize and transition from freely circulating to a fixture within the local ECM [42]. Vitronectin has been found to be upregulated in the serum of patients with early-stage DR and involved in diabetes-induced angiogenesis that occurs in late-stage DR [43,44]. Our study showed higher levels of glycosylation of vitronectin at site N-86 in DR patients. Hemopexin is an acute phase protein that binds to free heme to prevent its aberrant oxidative function in circulation [45]. However, hemopexin has been shown to have toxic protease activity that can lead to kidney damage reflected in diabetic nephropathy [34].

While the main function of plasminogen is to cleave fibrin to prevent blood coagulation, it also possesses anti-angiogenic properties that are enhanced by glycosylation [46,47]. Our study revealed that plasminogen is less glycosylated at site S-384 in the serum of DR patients. This glycosite is within the Kringle 4 fragment of plasminogen, which is the most potent angiostatin of this protein [46].

A glycopeptide of apolipoprotein C-III (APOC3), a known pro-inflammatory mediator [48], showed the most enriched glycosylation in DR. Increased levels of APOC3 in serum, plasma, and vitreous fluids have been associated with the occurrence and the severity of many metabolic disorders, including diabetes and DR [49,50,51]. Elevated serum APOC3 in diabetes has been shown to be a risk factor for DR due to its atherogenic properties [51]. APOC3 is known to be involved in a variety of inflammatory responses, including activation of endothelial cells and monocyte recruitment [52,53]. Di-sialylated APOC3 is associated with improved lipids in diabetes [54]. We also found that mono and di-sialylated glycoproteins were increased in the DR patients. This is in agreement with current literature that demonstrates the direct relationship of sialylation to both the onset of diabetes and the severity and degree of DR [55].

## 5. Conclusions

In conclusion, this study identified significant alterations in 15 serum glycopeptides belonging to 11 glycoproteins in DR patients. Many of these glycoproteins have been previously tied to vascular complications of diabetes, such as diabetic retinopathy or nephropathy. Considering the significant role these circulating glycoproteins have in chronic low-grade inflammation, these molecules are ideal candidates for future studies to discover precise molecular mechanisms and glycoproteomic-based therapeutics for DR. While the small sample size is the main limitation of our study, this in-depth analysis opens the door to study glycoproteomic alterations in DR using a larger cohort.

## Figures and Tables

**Table 1:** Patient demographics for subjects with and without diabetic retinopathy (DR).

Patient Characteristics	Patients Without DR	Patients With DR
Subjects	*N* = 13	*N* = 10
Gender (F/M)	8/5	7/3
Race (B/W)	5/8	6/4
Age (years)	69.25 ± 11.17	71.04 ± 12.15
Duration of Disease (years)	8.53 ± 2.83	12.81 ± 3.96
Stage of DR (NPDR/PDR)	-	4/6
Hypertension (Y/N)	6/7	7/3

**Table 2:** Most abundant glycopeptides detected in human serum.

Accession ID	Gene Symbol	Protein Name	Glycopeptide	Glycan Structure	Average PSM
P02790	HPX	Hemopexin	SWPAVGN^187^CSSALRW	1.1.0.1	17.27
P02765	AHSG	Alpha-2-HS-glycoprotein	TVVQPS^346^VGAAAGPVVPPCPGRI	1.1.0.1	16.85
P02790	HPX	Hemopexin	K.ALPQPQN^453^VTSLLGCTH-	4.5.0.2	15.60
P02765	AHSG	Alpha-2-HS-glycoprotein	K.VCQDCPLLAPLN^156^DTRV	4.5.0.2	10.38
P02790	HPX	Hemopexin	R.GHGHRNGTGHGN^246^STHHGPEYMRC	4.5.0.2	10.02
P19827	ITIH1	Inter-alpha-trypsin inhibitor heavy chain H1	R.TFVLSALQPSPT^653^HSSSNTQRL	1.1.0.1	8.18
P02765	AHSG	Alpha-2-HS-glycoprotein	K.AALAAFNAQNN^176^GSNFQLEEISRA	4.5.0.2	7.03
P08603	CFH	Complement factor H	K.IPCSQPPQIEHGTIN^882^SSRS	4.5.0.2	6.55
P02790	HPX	Hemopexin	R.GHGHRN^240^GTGHGNSTHHGPEYMRC	4.5.0.2	6.39
P00450	CP	Ceruloplasmin	AGLQAFFQVQECN^358^KS	4.5.0.2	6.14
P02790	HPX	Hemopexin	SWPAVGN^187^CSSALRW	4.5.1.2	5.75
P00738	HP	Haptoglobin	VVLHPN^241^YSQVDIGLIKL	4.5.0.2	5.70
Q14624	ITIH4	Inter-alpha-trypsin inhibitor heavy chain H4	LAILPASAPPATSNPDPAVS^709^RV	3.3.2.2	5.59
P0C0L5	C4B	Complement C4-B	GLN^1328^VTLSSTGRN	4.5.0.2	5.55
P00450	CP	Ceruloplasmin	EN^397^LTAPGSDSAVFFEQGTTRI	4.5.0.2	5.39
P00734	F2	Prothrombin	YPHKPEIN^143^STTHPGADLQENFCRN	4.5.0.2	5.35
P19827	ITIH1	Inter-alpha-trypsin inhibitor heavy chain H1	RTFVLSALQPS^651^PTHSSSNTQRL	1.1.0.1	5.07
P02765	AHSG	Alpha-2-HS-glycoprotein	TVVQPS^346^VGAAAGPVVPPCPGRI	1.1.0.2	4.99
P02765	AHSG	Alpha-2-HS-glycoprotein	KVCQDCPLLAPLN^156^DTRV	4.5.0.2	4.81
P05155	SERPING1	Plasma protease C1 inhibitor	VGQLQLSHN^352^LSLVILVPQNLKH	4.5.0.2	4.57
P00450	CP	Ceruloplasmin	EHEGAIYPDN^138^TTDFQRA	4.5.0.2	4.54
P02749	APOH	Beta-2-glycoprotein 1	VYKPSAGN^162^NSLYRD	4.5.0.2	4.54
P05546	SERPIND1	Heparin cofactor 2	N^49^LSMPLLPADFHKE	4.5.0.2	4.49
P02790	HPX	Hemopexin	SWPAVGN^187^CSSALRW	4.5.0.2	4.49
P02790	HPX	Hemopexin	ALPQPQN^453^VTSLLGCTH-	4.5.0.1	4.47
P01008	SERPINC1	Antithrombin-III	SLTFN^187^ETYQDISELVYGAKL	4.5.0.2	4.21
P00734	F2	Prothrombin	SRYPHKPEIN^143^STTHPGADLQENFCRN	4.5.0.2	3.88
P0C0L5	C4B	Complement C4-B	FSDGLESN^226^SSTQFEVKK	2.9.0.0	3.59
P02790	HPX	Hemopexin	CSDGWSFDATTLDDN^64^GTMLFFKG	4.5.0.2	3.33
P01008	SERPINC1	Antithrombin-III	LGACN^128^DTLQQLMEVFKF	4.5.0.2	3.25
P02790	HPX	Hemopexin	ALPQPQN^453^VTSLLGCTH-	4.5.0.2	3.17
P02790	HPX	Hemopexin	GHGHRNGTGHGN^246^STHHGPEYMRC	4.5.0.2	3.15
P00450	CP	Ceruloplasmin	EHEGAIYPDN^138^TTDFQRA	4.5.1.2	3.10
P00450	CP	Ceruloplasmin	ELHHLQEQN^762^VSNAFLDKG	4.5.0.2	3.10
P01019	AGT	Angiotensinogen	VYIHPFHLVIHN^47^ESTCEQLAKA	4.5.0.2	2.99
P01042	KNG1	Kininogen-1	FSVATQTCQITPAEGPVVT^137^AQYDCLGCVHPISTQSPDLEPILRH	1.1.0.1	2.98
P00747	PLG	Plasminogen	GNVAVTVSGHTCQHWSAQTPHTHN^308^RT	4.5.0.2	2.84
P19827	ITIH1	Inter-alpha-trypsin inhibitor heavy chain H1	TFVLSALQPSPTHSS^656^SNTQRL	1.1.0.1	2.49
P02656	APOC3	Apolipoprotein C-III	DKFSEFWDLDPEVRPT^94^SAVAA-	1.1.0.1	2.46
P00450	CP	Ceruloplasmin	ELHHLQEQN^762^VSNAFLDKGEFYIGSKY	4.5.0.2	2.44
P04196	HRG	Histidine-rich glycoprotein	R.VIDFN^125^CTTSSVSSALANTKD	4.5.0.1	2.35
P04114	APOB	Apolipoprotein B-100	FVEGSHN^3411^STVSLTTKN	4.5.0.1	2.34
P19823	ITIH2	Inter-alpha-trypsin inhibitor heavy chain H2	GAFISN^118^FSMTVDGKT	4.5.0.2	2.24
P02656	APOC3	Apolipoprotein C-III	FSEFWDLDPEVRPT^94^SAVAA-	1.1.0.1	2.22
P02749	APOH	Beta-2-glycoprotein 1	LGN^253^WSAMPSCKA	4.5.0.2	2.09
P04114	APOB	Apolipoprotein B-100	FVEGSHN^3411^STVSLTTKN	3.5.0.1	2.02
P00748	F12	Coagulation factor XII	RN^433^HSCEPCQTLAVRS	4.5.0.2	2.00
Q5JWR5	DOPEY1	Protein dopey-1	KALET^86^YEIIFKI	1.1.0.1	1.92
P02765	AHSG	Alpha-2-HS-glycoprotein	TVVQPS^346^VGAAAGPVVPPCPGRI	1.2.1.0	1.89

Glycan Structure: Numerical values respective to monosaccharides: N-acetylhexosamine (HexNAc), hexose (Hex), fucose (Fuc), and N-acetylneuraminic acid (NeuAc); Average PSM: Average peptide spectral matches; Red color indicates site of glycosylation.

**Table 3:** Significant alterations in the serum glycoproteome of patients with diabetic retinopathy.

Accession ID	Symbol	Protein Name	Glycopeptide(s)	Glycan	Glycan Structure	Glycan Mass	Fold Change DR	*p*-Value
**Upregulated**								
P02656	APOC3	Apolipoprotein C-III	FSEFWDLDPEVRPT^94^SAVAA.	HexNAc(1)Hex(1)NeuAc(2)	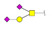	947.323	3.210	0.0002
P09871	C1S	Complement C1s subcomponent	NCGVN^174^CSGDVFTALIGEIASPNYPKPYPENSR.C	HexNAc(4)Hex(5)NeuAc(2)	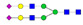	2204.772	2.858	0.0007
Q92954	PRG4	Proteoglycan 4	TITTTEIMNKPEET^1004^AKPK.D	HexNAc(2)Hex(2)NeuAc(2)	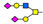	1312.455	2.210	0.0133
P04004	VTN	Vitronectin	NN^86^ATVHEQVGGPSLTSDLQAQSK.G	HexNAc(4)Hex(5)NeuAc(2)	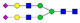	2204.772	2.104	0.0482
P01008	SERPINC1	Antithrombin-III	LGACN^128^DTLQQLMEVFK.F	HexNAc(4)Hex(5)NeuAc(2)	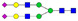	2204.772	1.789	0.0026
**Downregulated**								
P02751	FN1	Fibronectin	RHEEGHMLN^542^CTCFGQGR.G	HexNAc(4)Hex(5)NeuAc(1)	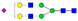	1913.677	0.069	7.43 × 10^−6^
P02751	FN1	Fibronectin	TTPPTT^2156^ATPIR.H	HexNAc(1)Hex(1)NeuAc(1)	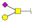	656.228	0.107	0.0007
P0C0L5	C4B	Complement C4-B	GLN^1328^VTLSSTGR.N	HexNAc(4)Hex(5)NeuAc(2)	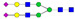	2204.772	0.185	7.25 × 10^−10^
P0C0L5	C4B	Complement C4-B	FSDGLESN^226^SSTQFEVK.K	HexNAc(2)Hex(9)	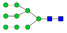	1864.634	0.207	1.53 × 10^−6^
P02765	AHSG	Alpha-2-HS-glycoprotein	LGGAEVAVTCTVFQTQPVTSQPQPEGANEAVPTPVVDPDAPPSPPLGAPGLPPAGSPPDS^297^HVLLAAPPGHQLHR.A	HexNAc(2)Hex(2)NeuAc(2)	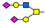	1312.455	0.328	0.0331
P02765	AHSG	Alpha-2-HS-glycoprotein	KVCQDCPLLAPLN^156^DTR.V	HexNAc(4)Hex(5)NeuAc(2)	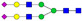	2204.772	0.641	0.0445
P02790	HPX	Hemopexin	GHGHRN^240^GTGHGNSTHHGPEYMR.C	HexNAc(4)Hex(5)NeuAc(2)	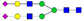	2204.772	0.256	0.0021
P02790	HPX	Hemopexin	GHGHRNGTGHGN^246^STHHGPEYMR.C	HexNAc(4)Hex(5)NeuAc(2)	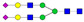	2204.772	0.639	0.0046
P19827	ITIH1	Inter-alpha-trypsin inhibitor heavy chain H1	TFVLSALQPSPTHSS^656^SNTQR.L	HexNAc(1)Hex(1)NeuAc(1)	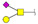	656.228	0.193	5.83 × 10^−5^
P00747	PLG	Plasminogen	IPSCDSSPVSTEQLAPTAPPELTPVVQDCYHGDGQS^384^YR.G	HexNAc(1)Hex(1)NeuAc(1)	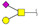	656.228	0.468	0.0374

Fold Change DR: Detection in diabetic retinopathy patients as compared to diabetic patients without DR; Red color indicates site of glycosylation.
